# Tumor Lysis Syndrome Triggered by Acalabrutinib in Chronic Lymphocytic Leukemia (CLL): Diagnostic and Therapeutic Implications

**DOI:** 10.7759/cureus.93185

**Published:** 2025-09-25

**Authors:** Monsif Fadi, Fatima Azzahra Lahlou, Yasmine El Ouafa, Hafsa Chahdi, Nouama Bouanani

**Affiliations:** 1 Hematology and Medical Oncology, Centre d'Études Doctorales - CeDOC, Mohammed VI University of Sciences and Health (UM6SS), Casablanca, MAR; 2 Oncopathology, Cancer Biology and Environment Laboratory, Mohammed VI Faculty of Medicine, Mohammed VI University of Sciences and Health (UM6SS), Casablanca, MAR; 3 Biochemistry, Faculty of Medicine, Mohamed VI University of Health Sciences (UM6SS), Casablanca, MAR; 4 Nutrition, Centre d'Études Doctorales - CeDOC, Mohammed VI University of Sciences and Health (UM6SS), Casablanca, MAR; 5 Interdisciplinary Laboratory of Biotechnology and Health, Mohammed VI University of Sciences and Health (UM6SS), Casablanca, MAR; 6 Pathology, Cheikh Khalifa International University Hospital, Mohammed VI University of Health Sciences, Casablanca, MAR; 7 Hematology, Faculty of Medicine, Mohammed VI University of Health Sciences (UM6SS), Casablanca, MAR

**Keywords:** bruton’s tyrosine kinase, bruton’s tyrosine kinase inhibitor, chronic lymphocytic leukemia (cll), immunophenotype, tumor-lysis syndrome

## Abstract

Tumor lysis syndrome (TLS) is an uncommon but potentially life-threatening oncologic emergency, typically associated with highly proliferative hematologic malignancies. It is rarely encountered in chronic lymphocytic leukemia (CLL), a generally indolent B-cell neoplasm. We report a unique case of therapy-induced TLS in a patient newly diagnosed with CLL, following the initiation of acalabrutinib, a Bruton’s tyrosine kinase (BTK) inhibitor. The patient presented with marked hyperleukocytosis, severe thrombocytopenia, and acute kidney injury at diagnosis. Immunophenotyping and histopathological evaluation confirmed the diagnosis of CLL, supported by a Matutes score of 4/5 and characteristic CD5+/CD23+/weak CD20 expression. Cytogenetic analysis revealed no high-risk abnormalities. Following acalabrutinib initiation, the patient rapidly developed metabolic disturbances, including severe hyperuricemia, hyperphosphatemia, hyperkalemia, hypocalcemia, and worsening renal function, consistent with laboratory and clinical TLS. Prompt management with intravenous hydration, Rasburicase, calcium supplementation, and potassium-lowering agents led to gradual metabolic correction. This case highlights the importance of recognizing the potential for TLS even in slow-growing hematologic malignancies like CLL, especially in patients with high tumor burden or compromised renal function. Clinicians must maintain a high index of suspicion and initiate appropriate prophylactic measures when starting targeted therapies, particularly BTK inhibitors, to prevent life-threatening complications.

## Introduction

Chronic lymphocytic leukemia (CLL) is a hematologic malignancy characterized by a monoclonal expansion of mature B lymphocytes, leading to infiltration of the bone marrow, peripheral blood, and occasionally lymph nodes. It primarily affects elderly individuals and is typically associated with a slow disease course, contributing to prolonged survival in many patients. CLL arises from a CD5+ B lymphocyte expressing low levels of surface membrane immunoglobulin (smIg), a unique light chain, and the markers CD79b, CD20, and CD23. Disease heterogeneity is attributed to both intrinsic factors - such as genetic and epigenetic alterations - and extrinsic influences from the tumor microenvironment (TME), which modulate signaling pathways [1]. Among the major oncologic emergencies, tumor lysis syndrome (TLS) stands out as a critical metabolic complication. It results from rapid tumor cell lysis, whether spontaneous or therapy-induced, leading to the release of intracellular components and subsequent metabolic abnormalities, including hyperuricemia, hyperkalemia, hyperphosphatemia, and hypocalcemia. These alterations may provoke acute renal failure, cardiac arrhythmias, seizures, and even death [2,3]. Though TLS is infrequent, the lack of preventive treatment can lead to significantly worse clinical outcomes [2]. TLS is rarely observed in indolent hematologic malignancies such as CLL, low-grade lymphomas, and multiple myeloma [4]. If TLS is not promptly managed, it can lead to severe complications, such as acute kidney failure, cardiac arrhythmias, neurological issues, or even seizures, and in the most extreme cases, death [5]. Furthermore, the management of TLS in patients treated with acalabrutinib presents specific clinical challenges. Preventive and therapeutic strategies, such as aggressive hydration, electrolyte monitoring, and the use of agents like Rasburicase, are well established in the context of conventional chemotherapy. However, their efficacy and safety in the setting of targeted therapies, particularly Bruton’s tyrosine kinase inhibitors (BTKis), require further rigorous evaluation [6].

## Case presentation

A 72-year-old patient with CLL was admitted for a rapid deterioration of his clinical condition in the context of TLS. On clinical examination, he had significant lymphadenopathy (cervical, axillary, and inguinal), NYHA class II exertional dyspnea, chest pain, and radiological findings consistent with pneumonia. Transthoracic echocardiography revealed a small pericardial effusion with preserved left ventricular ejection fraction (72 %). In addition, diffuse lymphadenopathy, pulmonary infiltrates, and acute renal failure were also noted, findings consistent with TLS.

The initial laboratory diagnostic revealed marked hyperleukocytosis (11,540/mm^3^) with 98 % blasts and 2 % neutrophils, along with severe thrombocytopenia (35,000/mm^3^). CRP was 12.4 mg/L, urea 67 mg/dL, and serum creatinine 15 mg/dL, indicating acute renal failure. Uric acid was elevated at 6.4 mg/dL, whereas procalcitonin was normal (0.20 ng/mL) and NT-proBNP was 1334 pg/mL. Immunophenotyping identified a monoclonal B-cell population strongly expressing CD19, CD5, CD23, and CD43, with positivity for FMC7 and weak expression of CD79b and CD20; a low-intensity expression of lambda light chains confirmed the monoclonality of these cells. This profile (CD5+/CD23+/weak CD20) is typical of CLL, with CD5 and CD23 positivity being particularly distinctive. The Matutes score was 4/5, strongly supporting the CLL diagnosis (this score is commonly used to distinguish CLL from other B-cell lymphoproliferations). The reduced expression of CD20 and CD79b is also consistent with the expected immunophenotype in CLL. Cytogenetic analysis by fluorescence in situ hybridization (FISH) on interphase and metaphase cells showed no abnormalities at the examined loci: specifically, no deletion of ATM (11q22) or of DLEU (13q14), nor alteration of TP53 (17p13) was detected. Likewise, no trisomy of chromosome 12 was found. The absence of these common cytogenetic abnormalities suggests a favorable genetic profile associated with a better prognosis, notably reflecting the intact TP53 gene and the lack of deleterious deletions.

**Figure 1:**
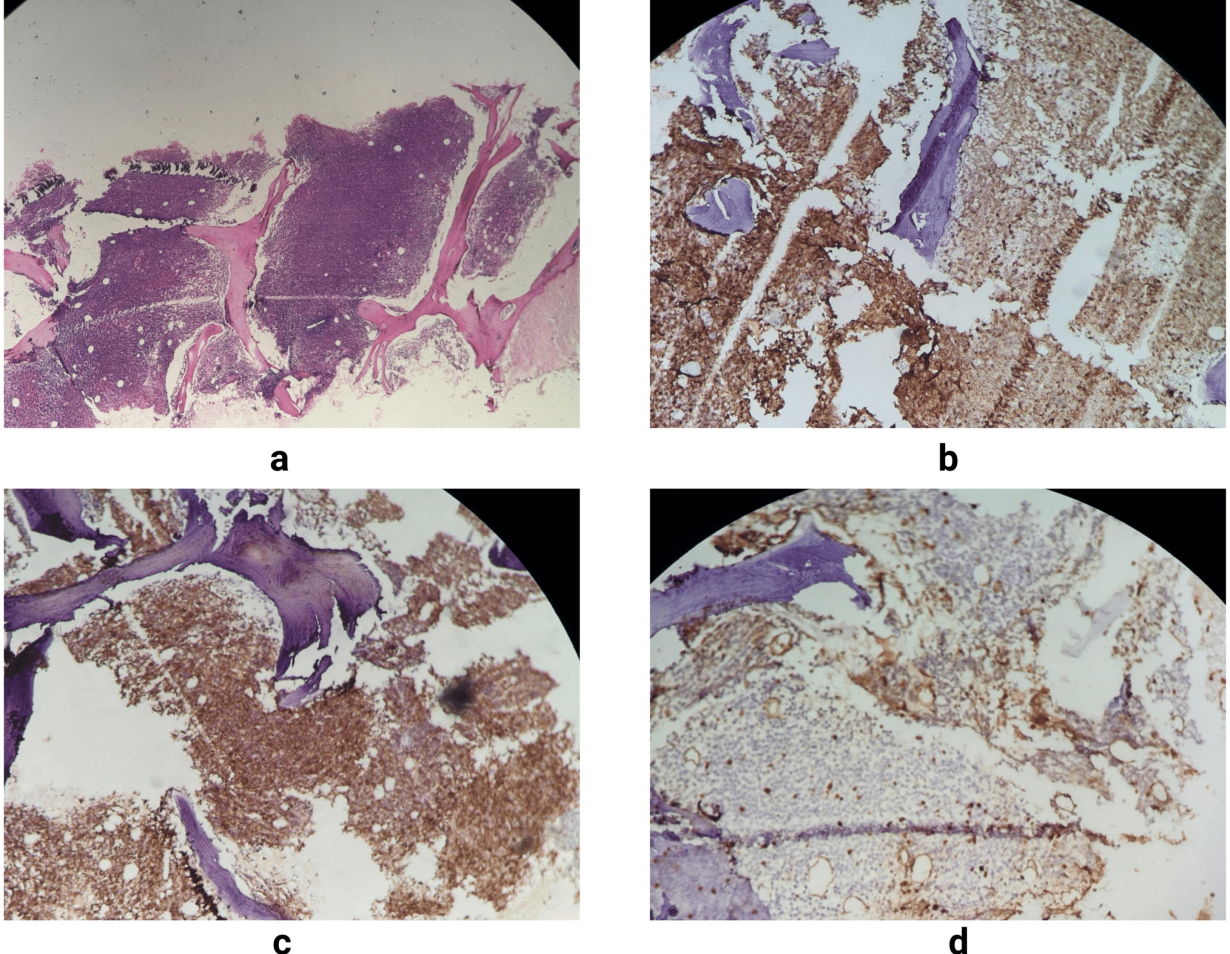
Bone marrow biopsy showing diffuse infiltration by small lymphocytes with strong CD20 (a) expression, aberrant co-expression of CD5 (b), and diffuse positivity for CD23 (c), confirming the diagnosis of chronic lymphocytic leukemia. The low Ki67 (d) proliferation index Histological sections demonstrate diffuse lymphoid infiltration with strong membranous expression of CD20 (a), confirming the B-cell nature of the proliferation. Aberrant co-expression of CD5 is observed (b), which is a characteristic immunophenotypic finding in CLL. Immunohistochemistry further reveals diffuse positivity for CD23 (c), supporting the diagnosis. The proliferation index assessed by Ki67 is low (d), estimated at approximately 15%, indicating limited mitotic activity. Taken together, these immunophenotypic and histopathological features, in correlation with the clinical and biological context, confirm the diagnosis of CLL with nasopharyngeal involvement.

Histopathological examination of the bone marrow biopsy revealed a diffuse infiltration by a monomorphic population of small mature lymphocytes, suggestive of a lymphoproliferative disorder. Immunohistochemistry demonstrated strong and diffuse membranous expression of CD20 on tumor cells, confirming their B-cell lineage, along with aberrant expression of CD5, establishing the typical immunophenotype of CLL. Co-expression of CD20 (a) and CD5 (b) supported the diagnosis and highlighted the atypical phenotype of malignant B cells in this entity. Moreover, CD23 (c) was strongly and diffusely expressed, particularly in areas with dense tumor cell infiltration, further supporting the diagnosis, as CD23 is a key differentiating marker from other B-cell lymphomas. The proliferation index assessed by Ki67 (d) staining was low (<20 %), with a few scattered positive cells, reflecting the indolent, low-grade nature of the disease. Serum protein electrophoresis revealed hypogammaglobulinemia, characterized by reduced gamma (6.1 %) and beta-2 (2.0 %) fractions. These findings are consistent with CLL and reflect impaired immunoglobulin production, potentially compromising immune defense (Figure 1).

**Figure 2:**
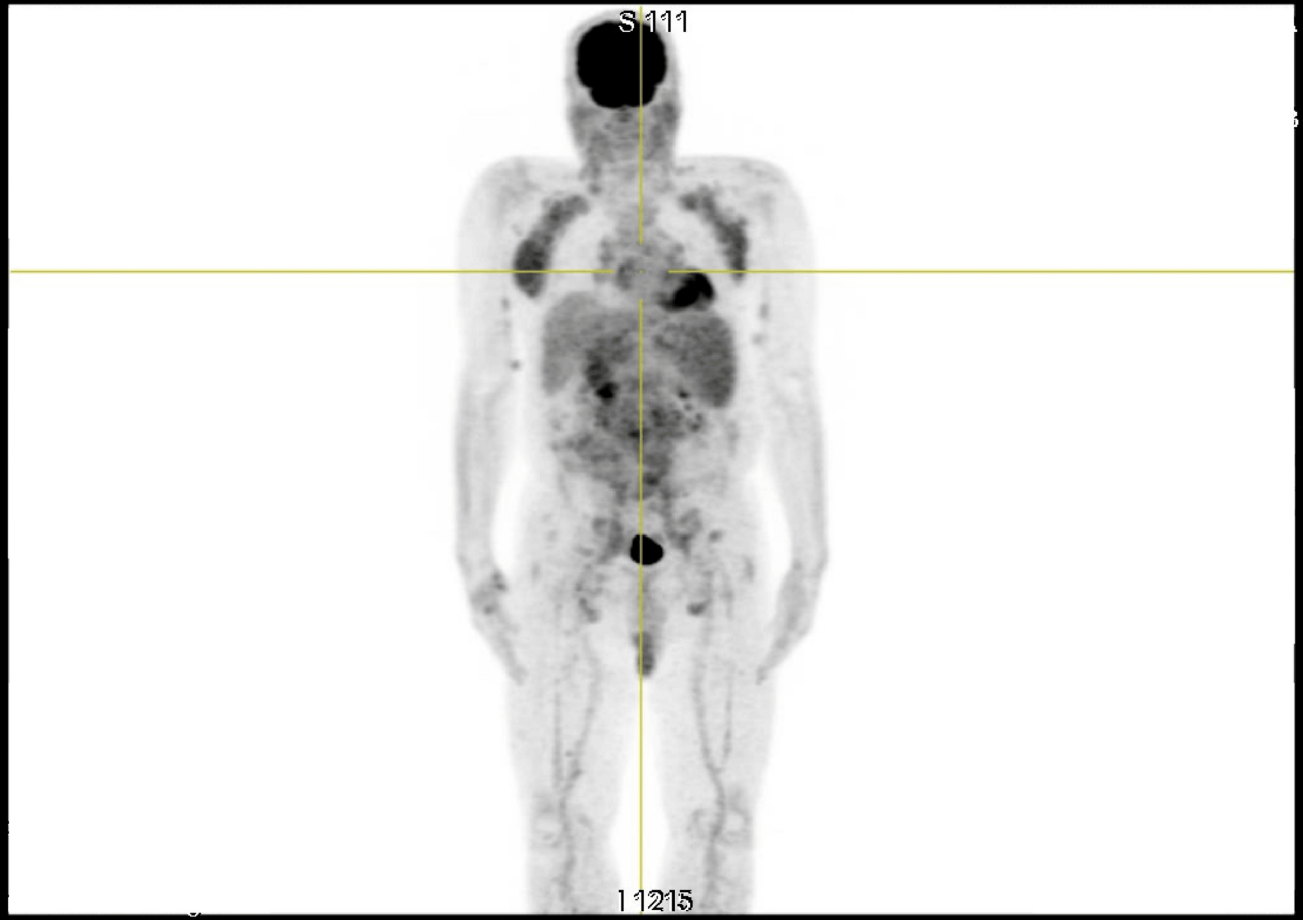
18F-FDG PET scan showing diffuse pathological hypermetabolism in the cervical, axillary, mediastinal, abdominal, pelvic, and inguinal lymph node regions Whole-body 18F-FDG PET/CT showing multiple hypermetabolic lymphadenopathies involving cervical, mediastinal, abdominal, and pelvic regions, associated with diffuse bone marrow uptake. The distribution and intensity of uptake are consistent with disseminated lymphoproliferative disease, supporting the diagnosis of CLL with systemic involvement.

The whole-body positron emission tomography (PET) scan demonstrated diffuse lymphadenopathy with predominantly mild to moderate fluorodeoxyglucose (FDG) uptake. In the cervico-thoracic region, bilateral lymphadenopathies involving the parotid, jugulocarotid, posterior cervical, and supraclavicular chains showed mild hypermetabolism, with a maximum standardized uptake value (SUVmax) of 2.87 on the right and 3.07 on the left. Axillary and pectoral lymph nodes were also mildly hypermetabolic, with SUVmax values of 4.36 on the right and 4.07 on the left. Multiple mediastinal lymph node stations exhibited low-grade FDG uptake, including prevascular (station 3A, SUVmax 2.21), right paratracheal (stations 2R and 4R, SUVmax 2.26), para-aortic (station 6, SUVmax 2.11), left lower paratracheal and subaortic (stations 4L and 5, SUVmax 2.81), subcarinal (station 7, SUVmax 3.06), and bilateral hilar nodes (stations 10R and 10L, SUVmax 2.70 and 2.64, respectively). In the abdominopelvic region, a bulky, moderately hypermetabolic lymph node conglomerate was observed, involving nearly all abdominal and pelvic nodal basins, with a SUVmax of 5.81. This process extended into the bilateral iliac chains (SUVmax 3.16 on the right and 3.57 on the left) and bilateral inguinal regions (SUVmax 2.68 on the right and 3.57 on the left). Hepatic FDG uptake was homogeneous (SUVmax 2.75), whereas splenic uptake was increased and suggestive of pathological involvement (SUVmax 3.81). Additionally, mildly hypermetabolic subcutaneous nodules were identified in the bilateral retroscapular and medial brachial regions (SUVmax ranging from 1.49 to 2.76). No abnormal metabolic activity was detected in the brain, bone marrow, or pulmonary parenchyma. A whole-body 18F-FDG PET scan was performed to assess disease dissemination. The scan showed diffuse pathological uptake across cervical, axillary, mediastinal, abdominal, pelvic, and inguinal lymph node regions. Increased tracer accumulation was also noted in the spleen and bone marrow. These findings support widespread nodal and medullary infiltration consistent with advanced-stage CLL. No suspicious focal extra-nodal lesions were identified, further confirming the diffuse nature of disease involvement (Figure 2).

**Figure 3:**
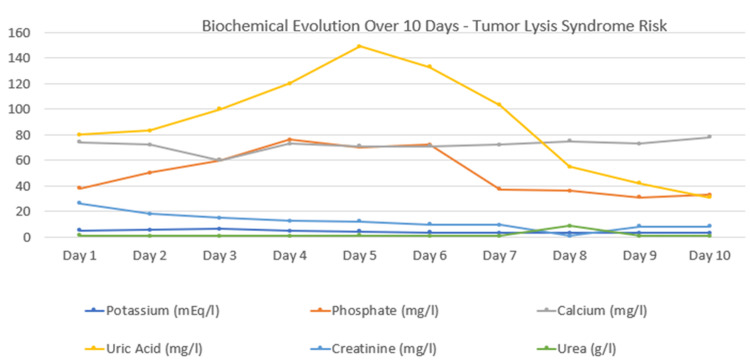
Graph showing the 10-day progression of key tumor lysis syndrome (TLS) biomarkers. Acalabrutinib was initiated on Day 1, coinciding with the early biochemical signs of TLS.

Uric acid levels were markedly elevated at 10 mg/dL, requiring prompt management to prevent uric acid nephropathy (Table 1). Serum potassium rose significantly, peaking at 6.2 mEq/L, indicating severe hyperkalemia, which was corrected using sodium polystyrene sulfonate to avoid arrhythmic complications. An electrocardiogram (ECG) was performed and showed no significant abnormalities suggestive of hyperkalemia-induced conduction disturbances. Serum phosphate was also elevated (3.8 mg/dL), raising concern for calcium phosphate precipitation and aggravation of TLS. Initial hypocalcemia (7.4 mg/dL) was managed with intravenous calcium supplementation, achieving a post-treatment level of 7.8 mg/dL. Acute kidney injury was evidenced by a markedly elevated serum creatinine of 2.63 mg/dL. Blood urea was moderately increased at 93 mg/dL, necessitating close monitoring of renal function and the implementation of supportive care measures. The patient received a treatment regimen that included acalabrutinib, a selective inhibitor of Bruton’s tyrosine kinase (BTK) commonly used in CLL and B-cell lymphomas. To manage the TLS, intravenous hydration was provided at 1 L/24 h to promote uric acid excretion and protect renal function. Sodium polystyrene sulfonate was given to control hyperkalemia, and Rasburicase was initiated to maintain uric acid at safe levels (Figure 3).

**Table 1:** Values of biochemical and hematological parameters at different time points (Day 1 to Day 10) Serial biological monitoring over 10 days demonstrated marked leukocytosis and lymphocytosis with peak values on day 3, consistent with the activity of CLL. Electrolyte disturbances included severe hyperkalemia (maximum 6.2 mEq/L on day 3) and progressive hyperphosphatemia, while calcium levels remained below the normal range. Uric acid rose significantly, peaking at 149 mg/L on day 5, indicating high tumor lysis activity. Renal function parameters revealed elevated creatinine and urea levels, consistent with acute kidney impairment, followed by gradual normalization after therapeutic intervention.

	Day 1	Day 2	Day 3	Day 4	Day 5	Day 6	Day 7	Day 8	Day 9	Day 10	Reference range
WBC (Elements/mm^3^)	7,000	7,100	11,540	5,100	5,000	5,520	9,990	12,060	12,600	9,240	3,800 - 10,000
Lymphocytes (Elements/mm^3^)	4,000	4,890	7,210	2,740	2,600	3,630	5,350	8,000	7,510	5,510	1,070 – 4,100
Potassium (mEq/l)	4.9	5.6	6.2	4.7	4.2	3.4	3.2	3	3.1	3.1	3.5 – 5.1
Phosphate (mg/dl)	3.8	5.0	6	7.6	7	7.2	3.7	3.6	3.1	3.3	2.3 – 4.7
Calcium (mg/dl)	7.4	7.2	6	7.3	7.1	7.1	7.2	7.5	7.3	7.8	8.5 – 10.1
Uric Acid (mg/dl)	8	8.3	10	12	14.9	13.3	10.3	5.5	4.2	3.1	2.6 – 7.2
Creatinine (mg/dl)	2.63	1.78	1.5	1.25	1.22	0.99	0.94	0.063	0.8	0.82	0.7 - 1.3
Urea (mg/dl)	93	76	67	92	95	81	61	845	54	63	15 – 45

## Discussion

TLS is a recognized emergency in both nephrology and oncology, requiring urgent and appropriate management, since without prompt intervention it can result in life-threatening complications [6,7]. TLS encompasses a group of metabolic abnormalities caused by the massive release of intracellular contents into the bloodstream following rapid tumor cell destruction [8]. In chronic hematologic malignancies, TLS is infrequently observed [9], as is also true for most solid tumors [10], primarily due to their typically slow proliferation rates and limited chemosensitivity. However, in highly proliferative and chemo‑sensitive tumors, TLS has been increasingly reported [11,12].

TLS generally presents in two forms: laboratory TLS, defined by the presence of at least two biochemical abnormalities hyperkalemia, hyperphosphatemia, hypocalcemia, or hyperuricemia, occurring within three days before to seven days after treatment initiation [13], and clinical TLS, which involves the same biochemical changes accompanied by acute kidney injury, seizures, or cardiac arrhythmias and constitutes a true medical emergency [14]. Laboratory criteria include hyperuricemia (≥ 476 μmol/L or ≥ 25 % increase), hyperkalemia (≥ 6.0 mmol/L or ≥ 25 % increase), hyperphosphatemia (≥ 1.45 mmol/L or ≥ 25 % increase), and hypocalcemia (≤ 1.75 mmol/L or ≥ 25 % decrease) [13], while clinical TLS is diagnosed when these criteria coincide with serum creatinine ≥ 1.5× upper limit of normal, seizures, or arrhythmias [15]. Although TLS is classically seen after cytotoxic therapy in acute lymphoblastic leukemia and Burkitt lymphoma, it can also occur with targeted agents or in unexpectedly sensitive tumors [8]. In this case, the team faced two critical challenges: CLL and therapy-induced TLS occurring despite no prior intensive chemotherapy. The onset of acute renal failure combined with severe electrolyte disturbances demanded an urgent, multidisciplinary response. Administration of acalabrutinib as a Bruton's tyrosine kinase inhibitor, alongside a rapid cytoreductive agent, likely precipitated extensive tumor cell lysis. Although acalabrutinib is generally well tolerated, its potent antitumor activity became hazardous in a patient with preexisting renal impairment, reducing the clearance of potassium and uric acid and thereby heightening the risks of hyperkalemia, hyperphosphatemia, and hyperuricemia. Renal dysfunction is a known predictor of worse outcomes in clinical TLS, underscoring the need for tailored management. Rigorous laboratory monitoring, prompt volume repletion, and timely use of uric acid-lowering therapies such as Rasburicase, combined with appropriate hydration, are essential to prevent life-threatening complications [16].

This case is particularly novel as TLS induced by acalabrutinib in CLL patients with renal insufficiency has been rarely reported, highlighting a potential risk even with agents traditionally considered low-risk for TLS [17]. It emphasizes the importance of proactive risk stratification, close monitoring of renal function and electrolytes, and individualized preventive strategies in patients receiving targeted therapies. By documenting this rare occurrence, this report provides practical guidance that may influence clinical decision-making, ensure patient safety, and inform best practices in the management of CLL.

## Conclusions

TLS is a life-threatening metabolic emergency typically associated with rapidly proliferating hematologic malignancies such as high-grade lymphomas and acute leukemias. However, this case demonstrates that TLS can also occur, albeit rarely, in patients with CLL, particularly in the presence of significant tumor burden and following initiation of cytoreductive therapy. This underscores the need for thorough pre-treatment risk assessment and implementation of appropriate prophylactic measures, even in patients with slower-growing malignancies like CLL. Physicians must be well-versed in recognizing the clinical features of TLS, its diagnostic criteria, and emergency management protocols, as prompt intervention is critical in avoiding serious or fatal outcomes.
